# Response to Letter regarding ”Motor and functional outcome of selective dorsal rhizotomy in children with spastic diplegia at 12 and 24 months of follow-up”

**DOI:** 10.1007/s00701-021-05058-w

**Published:** 2021-11-20

**Authors:** Matthias Schulz, Ulrich-Wilhelm Thomale

**Affiliations:** Pediatric Neurosurgery, Charité - Universitätsmedizin Berlin, Augustenburger Platz 1, 13353 Berlin, Germany

We thank the author for the letter about the published article from our institution about the motor and functional outcome after selective dorsal rhizotomy (SDR) and welcome the opportunity to further clarify aspects of the study’s methods.

With the treatment armamentarium for children with spastic diplegia SDR might play a significant role to reduce the amount of spasticity and thereby establishing the possibility for improved ambulation and overall motor function. Infantile cerebral palsy (ICP) constitutes a situation with a lifelong effect; therefore any treatment – including SDR – will ideally establish an live-long improvement. Treatment efficacy should therefore be measured after the longest possible follow-up. Several studies have demonstrated long-term efficacy of SDR to reduce spasticity [1, 2]. In this regard, the follow-up of 24 months in the presented study is at a lower range but still meaningful. We will continuously follow all treated patients beyond this reported two-years period and aim for markedly longer clinical observation into adolescence.

As Dr. Özden correctly pointed out, there are several confounding factors, which might influence the amount of spasticity after SDR. However, similar to the situation at the preoperative evaluation for possible SDR, no patients were under the effect of botulinum toxin injection (botox) at re-evaluation or did receive any botox during that time. All patients underwent standardized inpatient rehabilitation at specialized pediatric rehabilitation hospitals for several weeks postoperatively. For practical reasons and to avoid travel of the families the additional subsequent outpatient rehabilitation was allocated to the local physiotherapist at the family’s residential place.

The numerical data of the Modified Ashworth Scale (MAS, values of “1”, “1 + ”, “2”, “3” and “4”) as well of the Medical Research Council Scale (MRC, values of 0 to 5) represent data of an ordinal level. The median is the recommended measurement of central tendency of ordinal scales; mean and standard deviation represent data on a nominal level/scale and are better not applied to evaluate MAS and MRC scales. Likewise, the suggested paired Student’s t-test usually requires normal distribution of the variables and continuous dependent variables on an interval scale. Based on the ordinal level of the evaluated scales the non-parametric test (e.g. Wilcoxon matched-pair signed-rank test) was utilized for the presented data.

We agree with the statement, that ideally the measurements of the MAS and MRC should be collected by the same clinician in order to avoid observer bias. However, with a large patient cohort this ambition became less practical in our setting. In consideration of possible observer bias and to minimize this effect within the collected data all pre- and postoperative assessments were performed by a restricted group of clinicians of the multidisciplinary SDR team with extensive experience in taking care of children with spasticity and ICP.

In the context of other reports on the beneficious effects of SDR on spasticity and function and in respect to the discussed methodological limitations we are confident that the presented study provides further evidence of the merit of SDR in children with spastic diplegia.
